# QuickStats: Rates* of Emergency Department Visits Related to Mental Health Disorders Among Adults Aged ≥18 Years, by Disorder Category^†^ — National Hospital Ambulatory Medical Care Survey, United States, 2017–2019^§^

**DOI:** 10.15585/mmwr.mm7105a6

**Published:** 2022-02-04

**Figure:**
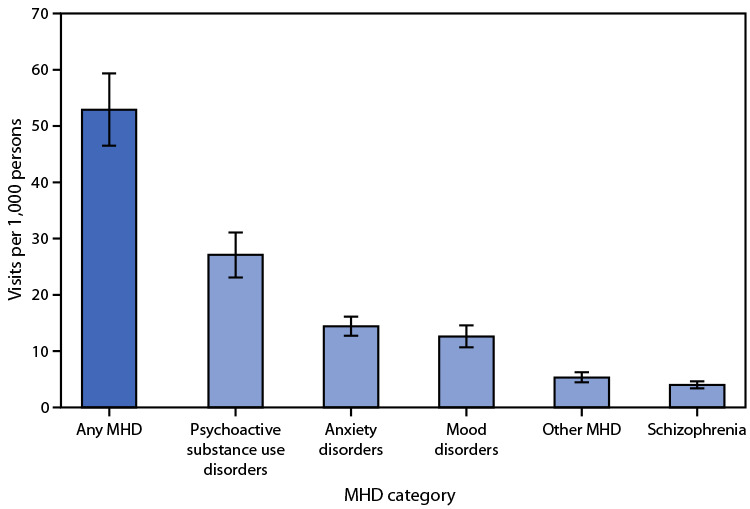
During 2017–2019, 52.9 ED visits per 1,000 persons were related to a diagnosed MHD in the United States per year. Approximately one half of mental health–related visits had a diagnosis of a psychoactive substance use disorder at a rate of 27.1 visits per 1,000 persons per year, followed by an anxiety, stress-related, or other nonpsychotic mental disorder (14.4), mood (affective) disorder (12.6), other MHD (5.3), and schizophrenia, schizotypal, delusional, or other nonmood psychotic disorder (4.0).

**Source:** National Center for Health Statistics. National Hospital Ambulatory Medical Care Survey, 2017–2019. https://www.cdc.gov/nchs/ahcd/ahcd_questionnaires.htm

For more information on this topic, CDC recommends the following link: https://www.cdc.gov/mentalhealth/tools-resources/individuals/index.htm.

